# Prospectively predicting 6-month risk for non-suicidal self-injury among adolescents after psychiatric hospitalization based on a predictive model

**DOI:** 10.3389/fpsyt.2024.1440808

**Published:** 2024-11-08

**Authors:** Wenjuan Zhu, Liping Cui, Huijie Zhang, Fang He, Min Li, Xufang Du, Xiaofen Fan, Wanling Li

**Affiliations:** ^1^ Shanxi Bethune Hospital, Shanxi Academy of Medical Sciences, Tongji Shanxi Hospital, Third Hospital of Shanxi Medical University, Taiyuan, China; ^2^ Nursing School, Shanxi Medical University, Taiyuan, China; ^3^ Third Hospital of Shanxi Medical University, Shanxi Bethune Hospital, Shanxi Academy of Medical Sciences, Tongji Shanxi Hospital, Taiyuan, China; ^4^ Tongji Hospital, Tongji Medical College, Huazhong University of Science and Technology, Wuhan, China

**Keywords:** logistic regression, predictive model, non-suicidal self-injury, adolescents, risk assessment model (RAM)

## Abstract

**Background:**

It is challenging to predict the occurrence of non-suicidal self-injury (NSSI) among adolescents over short periods. Moreover, the predictive value of indices for NSSI remains elusive. Thus, this study aimed to identify predictors of NSSI within 6 months among adolescents after psychiatric hospitalization by establishing a risk assessment model.

**Methods:**

A total of 632 high-risk participants were included in this study. The distribution characteristics of adolescent NSSI were initially assessed through a cross-sectional survey, following which risk factors were identified using logistic regression analysis. The risk score method was then used to construct a 6-month risk assessment model for NSSI. Lastly, the predictive effect of the model was evaluated by indicators such as the area under the receiver operating characteristic (ROC) curve and the positive predictive value.

**Results:**

After 6 months, 412 cases of NSSI were identified. According to the logistic regression model, the frequency of relapses, medication status, and NSSI history were identified as influencing factors. Higher scores on the Impulsive Behavior Scale and Pittsburgh Sleep Quality Index were associated with a higher risk of NSSI. Conversely, higher scores on the Pain and Belief Perception Scale were correlated with a lower risk of NSSI. Moreover, the area under the ROC curve for the predictive model was 0.9989, with a 95% confidence interval of (0.9979, 0.9999), highlighting its high predictive ability and accuracy. The predictive model was validated using 78 patients, yielding an area under the ROC curve of 0.9703 and a 95% confidence interval of (0.9167, 0.9999), demonstrating outstanding predictability.

**Conclusion:**

These results collectively showed that the predictive model could accurately predict adolescent NSSI. Thus, the model’s primary variables may be applied to predict the risk of NSSI in the clinical setting.

## 1 Background

Non-suicidal self-injury (NSSI) is defined as deliberate and intentional harm to one’s body without suicidal intent (1, 2) by cutting, hitting, scratching, and burning oneself (3). Mounting evidence indicates that the physiological growth of middle school students is accelerating during this developmental stage (4). Nevertheless, their psychological development frequently lags behind. During this period, adolescents have low psychological resilience, are susceptible to impulsive behaviors and excessive NSSI behaviors, and are unable to properly manage their negative emotions (5). Notably, NSSI not only causes severe physical and psychological injury to middle school students but also raises the risk of suicide among self-injurers, thereby imposing an additional burden on society and families. In 2018, 48,344 suicides were reported in the United States alone (6), with a key cause being the increase in NSSI behaviors (6, 7). Indeed, NSSI has become a substantial worldwide public health issue, especially among teenagers (8–10).

To date, numerous studies have examined the important components or predictors of NSSI. For instance, Gandhi et al. (11) described that the incidence of NSSI is the highest in 14–15-year-old individuals and progressively declines over time. Meanwhile, younger adolescents are at a higher risk of developing NSSI. Additionally, earlier studies (12) identified depression and a history of previous NSSI as risk factors for NSSI behavior in teenagers. NSSI may also be a result of familial or environmental dysfunction. Divorce or widowhood signifies a breakdown in family relationships, which exerts a significant negative influence on adolescents. Parental harsh punishment, low parental monitoring, and poor quality of attachment to parent predicted are associated with an increased risk of subsequent NSSI onset in adolescents, whereas positive parenting behaviors are associated with lower odds of NSSI onset during the following year (13). Therefore, the lack of functional family dynamics is a significant external contributor to NSSI behaviors in teenagers.

According to a previous study, NSSI is associated with impulsive tendencies and neurocognitive impulsivity (14). Notably, difficulties with impulse control (feeling out of control while distressed) were positively associated with NSSI history. These difficulties can differentiate between individuals with and without a history of NSSI (15). Patients with NSSI behaviors have impaired inhibitory control, behavioral disinhibition, and increased motor impulsivity. Based on previous studies, we speculate that impulsive behavior may be a predictor of NSSI behavior.

The non-linear association between sleep duration and NSSI in teenagers suggests that sleep patterns are linked to the patient’s NSSI status over the previous year (16). Poor sleep is associated with a higher risk of NSSI as a proxy for unfavorable emotion regulation. A study also found that current sleep patterns had a modest impact on the association between past and current NSSI behaviors. At the same time, a history of past NSSI could predict the occurrence of nightmares and suicide attempts. The strongest predictor of current NSSI was the recollection of past NSSI incidents, while current poor sleep was merely modestly associated with current NSSI behaviors. An additional indicator of current NSSI behaviors was experiencing nightmares within the last 6 months (17). Therefore, the predictive value of sleep for NSSI warrants further investigation.

Pain processing has emerged as a critical biological factor affecting the occurrence of NSSI. Indeed, diminished pain sensitivity and baseline opioid deficit were identified as possible risk factors for NSSI (18). However, clinical evidence for the association between pain sensitivity and NSSI is lacking, necessitating further exploration (18). Parallel mediation analyses have demonstrated that psychache independently mediated significant links between any type of teenage trauma and NSSI (19). Mental pain, a generally unbearable pain, modulates the occurrence of trauma and NSSI behavior in adolescents. Nevertheless, the correlation between pain processing and NSSI requires further investigation.

Despite extensive research conducted on NSSI, significant gaps remain. The majority of previous studies investigating NSSI examined the overall frequency of NSSI behaviors without considering the type of behavior or frequency of specific behaviors performed (20–22), oversights that may have potentially led to missing crucial information that could contribute to risk assessment. Consequently, only a few variables with satisfactory predictive power have been identified (23), most likely due to the limited variance in NSSI occurrence that can be explained by individual predictors. Variables that have been identified as predictive of NSSI behavior in young adults include depressive symptoms, anxiety symptoms, female gender, affective dysregulation, and ruminative cognitive styles (24–28).

In addition to examining predictors of NSSI, it is essential to identify potential processes or major risk factors that can assist doctors, parents, and community workers in making decisions on the prevention and management of NSSI. Based on these predictors, tailored psychological intervention measures or psychological care programs can be implemented. There is a pressing need to precisely identify those at high risk for future suicidal behavior. Therefore, new predictive methods or models are required to identify adolescents at high risk.

This study aimed to examine the 6-month risk for non-suicidal self-injury among adolescents after psychiatric hospitalization. Specifically, it sought to investigate the direct predictive effect on NSSI based on a predictive model. Given that NSSI may be impacted by general demographics, medication adherence, sleep quality, pain perception, impulsive behavior, etc., it is necessary to examine these variables. Moreover, these variables are readily accessible to medical personnel and easier to acquire than hematological indicators. The 6-month period following hospitalization is a crucial time point for patients. Consequently, the predictive model was based on the obtained data, and a confirmatory study was performed to validate its accuracy and predictive ability.

## 2 Methods

### 2.1 Participants

From January 2019 to July 2023, data on adolescents with NSSI hospitalized in the psychiatry department of a tertiary general hospital were collected. The inclusion criteria for participants were as follows: a) met the Diagnostic and Statistical Manual of Mental Disorders, Fifth Edition (DSM-5) criteria NSSI, b) aged 13–23 years, c) no gender restriction, d) hospitalized patients who could be followed up, and e) informed consent was obtained, with voluntary participation. This research adhered to the principle of voluntariness, respecting and safeguarding the privacy of research participants. Exclusion criteria for patients were as follows: a) strong suicidal ideation; b) present or prior history of schizophrenia, delusional illness, anxiety disorder, intellectual disability, and autism; c) history of psychoactive substance; and d) severe physical diseases. In this investigation, purposeful sampling was employed. A total of 632 patients met the inclusion criteria.

### 2.2 Measures

#### 2.2.1 Clinical observation index collection questionnaire

The questionnaire was derived from a review of relevant research, consultations with psychiatric professionals, and consideration of the hospital’s conditions, which included the following information: a) gender, b) age, c) educational attainment degree, d) marital status, e) whether the participant was an only child, f) family relationship status, g) medication adherence, h) NSSI history, and i) NSSI recurrence.

#### 2.2.2 Beck Scale for Suicide Ideation

The Beck Scale for Suicide Ideation [Beck Scale for Suicide Ideation-Chinese Version (BSI-CV)] compiled by Beck (29) was translated and reviewed by Li Xianyun et al. (30) and covered 19 items, with each item scored on a 3-point scale (0–2 points), yielding a total score ranging between 0 and 38 points. Greater scores reflect stronger suicidal ideation and suicide risk. Cronbach’s α coefficients for suicidal ideation in the last week and at its most severe point were 0.68 and 0.87, respectively, while the test–retest reliability coefficients were 0.64 and 0.76, respectively.

#### 2.2.3 Impulsive Behavior Scale BIS-11

The Barratt Impulsiveness Scale (BIS-11) was originally developed by Barratt in 1959 (31), and Zhou Liang adapted the Chinese version based on the revised 2006 Barratt Impulsiveness Scale (32). The scale consists of 26 items, with 11 items reverse-scored. It includes three subscales: attentional impulsivity, motor impulsivity, and non-planning impulsivity. Responses were rated on a 4-point Likert scale (1 to 4), with higher scores indicating greater levels of impulsivity. In this study, Cronbach’s α coefficient for the scale was 0.75.

#### 2.2.4 Pain Beliefs and Perceptions Inventory

The Pain Beliefs and Perceptions Inventory (PBPI) was designed by the American psychologist Williams et al. (33) to examine the impact of temporal changes on patients’ pain beliefs. The scale covers four dimensions: the perception that pain is inexplicable, the belief that it will persist, the conviction that the agony cannot be cured, and the sense of self-blame. Higher scores indicate stronger negative beliefs. Chinese scholars translated this scale in 2008. Cronbach’s α coefficient for the measured scale was 0.731 (34).

#### 2.2.5 Pittsburgh Sleep Quality Index

The Pittsburgh Sleep Quality Index (PSQI) was compiled in 1989 by Dr. Buysse, a psychiatrist at the University of Pittsburgh, USA (35). It consists of 19 self-evaluated items and five items rated by others. Each item is scored on a scale of 0 to 3, with a total score of 0 to 21. It consists of seven components, including subjective sleep quality, time taken to fall asleep, sleep duration, sleep efficiency, sleep disorders, use of sleep medications, and daytime functions, divided into the sum of 7-factor points. A total PSQI score of ≤7 indicates normal sleep, whereas a total score of >7 suggests sleep disorders. In terms of reliability, Cronbach’s α coefficient of the PSQI was 0.7962. In terms of validity, the correlation coefficient between the total scores from repeated tests was 0.8126.

### 2.3 Data collection

Prior to data collection, patients and their primary caregivers were informed of the study’s objective and relevance and willingly provided informed consent. Upon discharge from the psychiatry ward, all scales were assessed according to the physician’s order. Four registered nurses in the psychiatry unit, as well as two graduate students and two certified head nurses, performed the data collection. The objective and methodology of the study, data collection protocols, and quality control measures were covered during investigator training. Data were collected from the hospital’s medical record information system in strict accordance with operational criteria. Staff members engaged in mutual supervision to ensure that data collection was completed within the allotted time and in accordance with ethical standards and quality control requirements. The instructor conducted the final quality assurance. Every Friday afternoon, research team members actively raised issues encountered during data collection and addressed recurring issues. Patients in the psychiatry unit completed the surveys in accordance with a unified set of instructions. Those unfamiliar with the surveys or who experienced difficulty in writing were provided with detailed explanations. The questionnaires were promptly collected after completion, and the acquired data were stored by the project manager to prevent modifications. After discharge, patients were predominantly followed up via telephone. Following the completion of data collection, two data entry operators inputted and verified the data.

### 2.4 Statistical analysis

All statistical analyses were performed using SAS 9.4. Continuous variables conforming to a normal distribution were expressed as means and standard deviations and compared using independent-samples *t*-tests. Variables with a skewed distribution were presented as the median and interquartile ranges and compared using the Wilcoxon rank-sum test. Quantitative data were compared using either ANOVA or the Wilcoxon rank-sum test. Categorical data were described as frequencies and compared using the *χ* ^2^ test or Fisher’s exact test. In this study, logistic regression analysis was performed to analyze count variables and construct the predictive model. The model was validated using the Hosmer–Lemeshow goodness-of-fit test, and receiver operating characteristic (ROC) curve analysis was performed to assess the predictive capacity of the model.

## 3 Results

### 3.1 Patient characteristics

A total of 632 cases, aged between 13 and 23 years with an average age of 17.26 ± 2.31, were included in this study. Among them, 204 patients presented with first-time NSSI, representing 32.28% of all cases. The remaining 428 patients experienced recurrent NSSI, accounting for 67.72% of the cohort. Within 6 months after hospitalization, 412 cases developed NSSI, accounting for 65.19% of the cohort.

### 3.2 Single-factor analysis of NSSI risk within 6 months after hospitalization

Single-factor analysis was carried out on demographic characteristics, family relationships, medication adherence, self-injury history, hospitalization, Beck suicide ideation scores, BIS-11 scores, Pain Beliefs and Perceptions Inventory scores, and Pittsburgh Sleep Quality Index. Group comparison for categorical data was carried out using the *χ* ^2^ test, and the results revealed four statistically significant variables, namely, gender, medication adherence, and NSSI history and recurrence (*p* < 0.05). See **Table 1**.

**Table 1:** *χ* ^2^ test results of NSSI risk within 6 months after hospitalization.

Participant variables	Without NSSI n (%)	With NSSI n (%)	Statistical testing	*p*
**Gender**			18.658^*^	<0.001
Male = 1	32 (14.6)	124 (30.1)		
Female = 0	188 (85.5)	288 (69.9)		
**Education degree**				
Junior high school = 0	44 (20.0)	88 (21.4)	3.779^*^	0.286
High school = 1	96 (43.6)	196 (47.6)		
Junior college = 2	31 (14.1)	62 (15.0)		
Undergraduate and above = 3	49 (22.3)	66 (16.0)		
**Marriage status**			^△^	0.873
Unmarried = 0	217 (98.6)	407 (98.8)		
Married = 1	3 (1.4)	5 (1.2)		
**The only child**				
No = 0	135 (61.4)	239 (58.0)	0.668^*^	0.414
Yes = 1	85 (38.6)	173 (42.0)		
**Family relationship**			^△^	0.435
Discord = 0	3 (1.4)	8 (2.0)		
General = 1	91 (41.4)	149 (36.3)		
Harmony = 2	126 (57.3)	254 (61.7)		
**Regular medication**			16.577^*^	<0.001
No = 0	105 (47.7)	129 (31.3)		
Yes = 1	115 (52.3)	283 (68.7)		
**NSSI history**			55.429^*^	<0.001
No = 0	131 (59.5)	120 (29.1)		
Yes = 1	89 (40.5)	292 (70.9)		
**Recurrence**			76.547^*^	<0.001
No = 0	120 (54.6)	84 (20.4)		
Yes = 1	100 (45.5)	328 (79.6)		

NSSI, non-suicidal self-injury.

*: *χ* ^2^ test; △: Fisher exact probability method.

The *t*-test was used to compare quantitative data and identified five statistically significant variables associated with NSSI risk, namely, the number of relapses, the Beck suicidal ideation scale scores, the Impulsive Behavior Scale BIS-11 scores, Pain and Belief Perception Scale scores, and the Pittsburgh Sleep Quality Index (*p* < 0.05). See **Table 2**.

**Table 2:** *t*-Test results of NSSI within 6 months after hospitalization.

Variable	Whether NSSI occurred within 6 months	N	Mean	Standard deviation	Statistical testing	*p*
Age	No	220	17.19	2.249	0.57	0.570
	Yes	412	17.30	2.345		
The number of relapses	No	220	0.800	0.925	8.89	<0.001
	Yes	412	1.476	0.903		
Beck suicidal ideation scale	No	220	13.855	5.637	7.82	<0.001
	Yes	412	18.087	6.891		
Impulsive Behavior Scale BIS-11	No	220	83.27	5.2903	11.48	<0.001
	Yes	412	87.45	3.7721		
Pain and Belief Perception Scale	No	220	1.07	0.7022	46.57	<0.001
	Yes	412	-1.21	0.5127		
Pittsburgh Sleep Quality Index	No	219	5.25	0.9217	25.29	<0.001
	Yes	411	7.55	1.1682		

NSSI, non-suicidal self-injury.

### 3.3 Multivariate analysis of NSSI risk within 6 months after hospitalization

Furthermore, nine variables with statistical significance in the univariate analysis were incorporated in the binary logistic regression for stepwise regression analysis to account for confounding factors and further explore influencing factors. As summarized in **Table 3**, the number of relapses, medication adherence, NSSI history, impulsive behavior, pain, and belief perception scale scores, and the Pittsburgh Sleep Quality Index were included in the regression equation. Importantly, the logistic model identified the frequency of relapses, medication adherence, and NSSI history as factors influencing the outcomes. Among them, the risk of NSSI in patients with poor medication compliance was 4.00 times higher than that of patients with good medication compliance after 6 months. Similarly, the risk of NSSI in patients with a history of NSSI was 5.807 times that of patients without a history of NSSI after 6 months. Notably, for each additional recurrence, the risk of NSSI increased by 2.150 times. In addition, impulsive behavior, Pain and Belief Perception Scale scores, and sleep quality index were correlated with the predicted risk of NSSI. Specifically, the Impulsive Behavior Scale scores were positively correlated with NSSI risk after 6 months. Conversely, the Pain and Belief Perception Scale score was negatively correlated with NSSI risk after 6 months. Finally, the Pittsburgh Sleep Quality Index was positively correlated with NSSI risk after 6 months. See **Table 3**.

**Table 3:** Logistic stepwise regression analysis of NSSI risk within 6 months after hospitalization.

Variable	Estimated value	Standard deviation	Wald *χ* ^2^	*p*	*RR*	95% confidence interval	
Constant	−105.9	22.834	21.528	<0.0001			
Medication	1.386	0.697	3.957	0.047	4.000	1.021	15.676
Number of relapses	0.765	0.347	4.879	0.027	2.150	1.090	4.240
NSSI history	1.759	0.728	5.835	0.016	5.807	1.393	24.196
Impulsive Behavior Inventory BIS-11	1.043	0.247	17.792	<0.0001	2.837	1.748	4.607
Pain and Belief Perception Scale	−3.502	0.549	40.737	<0.0001	0.030	0.010	0.088
Pittsburgh Sleep Quality Index	1.574	0.376	17.525	<0.0001	4.826	2.310	10.084

The result of the Hosmer–Lemeshow test yielded a chi-square value of 0.5018 (*p* > 0.05), indicating that the model fits well.

NSSI, non-suicidal self-injury.

### 3.4 Nomogram construction for NSSI risk among adolescents within 6 months after hospitalization

**Figure 1:**
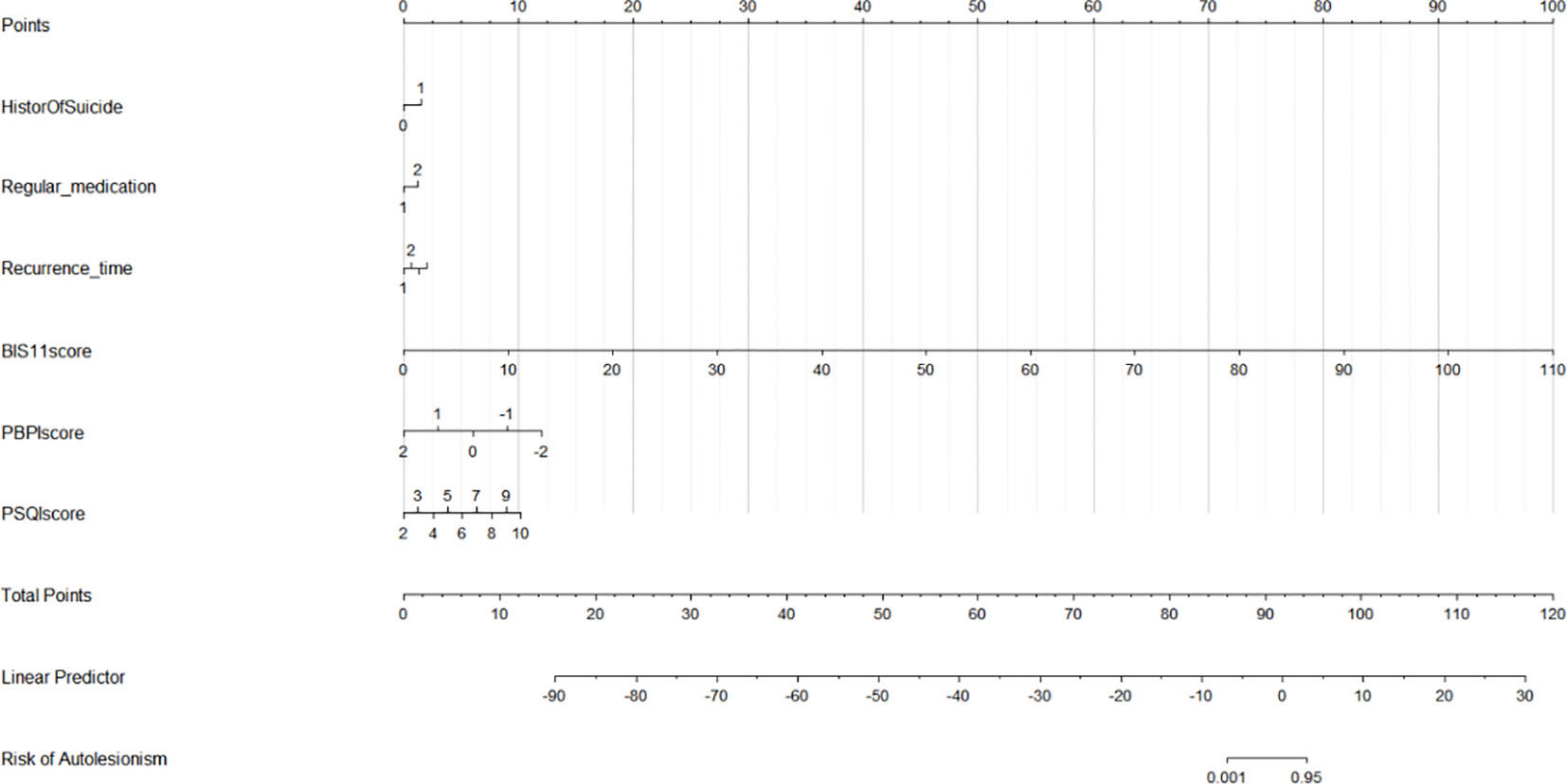
Nomogram.

The developed nomogram is illustrated in **Figure 1**, with each variable corresponding to a specific score. The overall score was determined by summing the individual scores. The projected likelihood of the total score reflects the risk of NSSI within 6 months after hospitalization among adolescents.

### 3.5 Validation of predictive models

**Figure 2:**
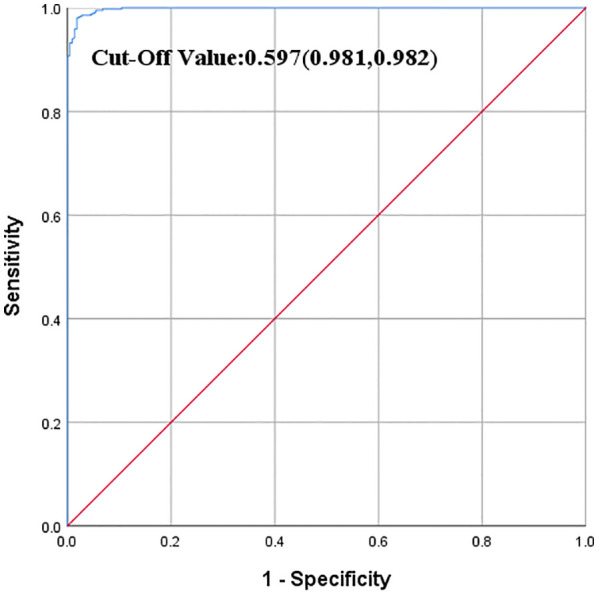
Results of the predictive model validating the original data.

This study employed the logistic regression model to construct an NSSI risk prediction model within 6 months after hospitalization. The result showed that the area under the ROC curve of the predictive model was 0.9989, with a 95% confidence interval of (0.9979, 0.9999) based on the original data. At a critical value of 0.597, the sensitivity, specificity, and Youden’s index were 0.981, 0.982, and 0.963, respectively. See **Figure 2**.

**Figure 3:**
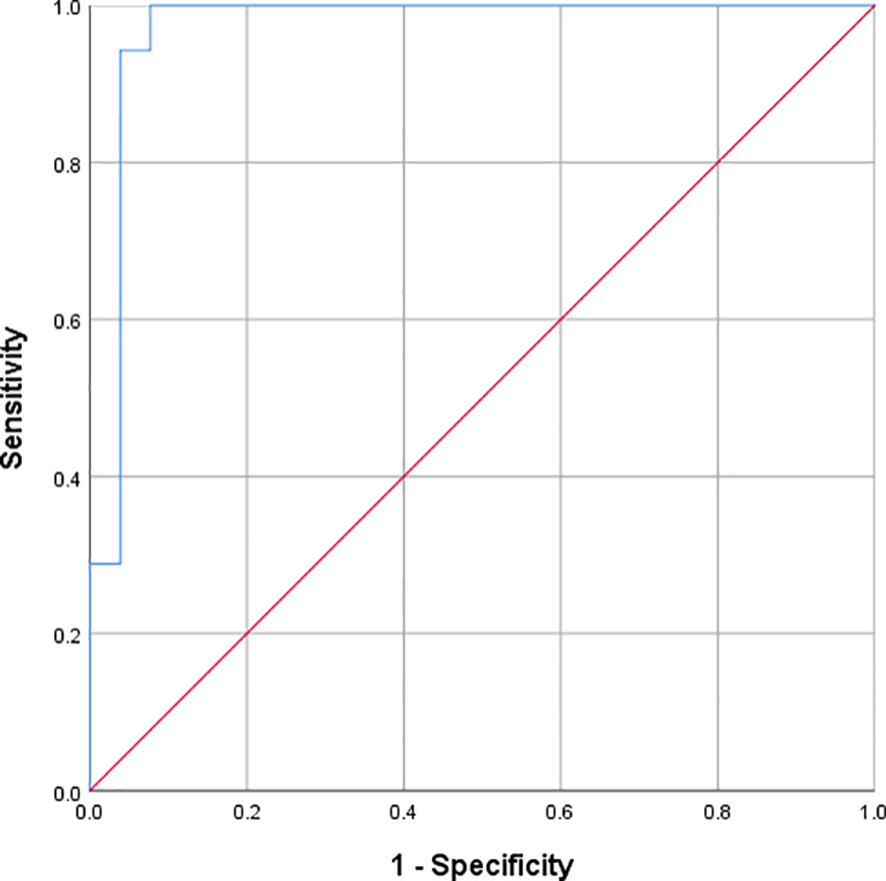
Validation of the predictive model for 78 cases within 6 months after hospitalization.

To evaluate the predictive ability of the model, 78 newly studied cases were included. Among them, 30 were male (38.46%) and 48 were female (61.54%). Their average age ranged from 13 to 22 years, with a mean of 17.51 ± 2.295 years. The area under the ROC curve was 0.9703 for these cases, with a 95% confidence interval of (0.9167, 0.9999). See **Figure 3**.

## 4 Discussion

This prospective study aimed to assess the 6-month risk for NSSI among adolescents hospitalized for psychiatric reasons. Specifically, the direct predictive effect on NSSI was explored using a predictive model. Moreover, baseline predictors of NSSI over a 6-month follow-up period were identified using multivariable models. Our findings conjointly revealed that adolescent NSSI is impacted not only by demographic variables such as gender and age but also by medication adherence, sleep index, pain perception, impulsive behavior, and other variables. While considering pain perception, medication compliance, and sleep quality pose challenges in risk recognition, a predictive model of NSSI was constructed and validated, highlighting its high predictive and testing abilities. This model can be promoted and implemented in clinical settings.

### 4.1 The prevalence of NSSI among adolescents is relatively high

The prevalence of NSSI in this study was 32.28%. Another study reported a self-injury detection rate of 33.7% among 3,600 middle school children (36). A research team previously conducted a meta-analysis on the prevalence of NSSI in teenagers and reported a global prevalence of 21% [Effect Size (ES) = 0.21, 95% CI (0.18, 0.25)] among teenagers.

However, as demonstrated in this study, the prevalence of NSSI is very high. This finding may be ascribed to the timing and location of the survey or the age range of participants. The mental health education system in China requires further improvement and development. The rehabilitation model should evolve from hospitalization to early prevention. In addition, the mental health service system in Europe, North America, and Oceania is relatively well-established, with adolescents accessing professional mental health education earlier (37, 38). Variations in the timing of investigation may also lead to differences in the incidence of NSSI. With socioeconomic development and increased academic pressure, adolescent mental health issues may become more prominent, leading teenagers to resort to extreme measures to cope with negative emotions. Consequently, NSSI has emerged as a global mental health issue that should be prioritized by researchers and clinical experts. On the one hand, it is essential to foster an atmosphere favorable to the healthy development of youth. On the other hand, it is vital to promote early screening for NSSI and implement prompt intervention methods in order to assist adolescents in developing effective coping strategies and avoiding NSSI.

### 4.2 Analysis of risk factors in the NSSI risk prediction model

#### 4.2.1 Increased recurrences increase the risk of NSSI post-hospitalization

Notably, recurrent NSSI has become one of the most prevalent characteristics associated with this behavior (39). According to longitudinal research on NSSI undertaken by Plener et al. (40), NSSI typically initiates during early to middle adolescence and recurs throughout the lifespan of individuals. A questionnaire survey at a university unveiled that the detection rate of two or more self-harming behaviors was 67.3% (41), with 8.8% of male participants employing over five methods for NSSI (41). According to the experience avoidance paradigm, the purpose of NSSI is to evade or escape undesirable experiences or behaviors. Chapman’s experience avoidance model posits that individuals with emotional management disorders may respond to triggering external events by resorting to self-injury as a coping strategy to escape painful emotional experiences (42).

NSSI has significant characteristics of behavioral addiction and is a mechanism for emotional venting. If individuals experience a reduction in negative emotions immediately after NSSI, the tendency to implement this behavior becomes stronger when the negative emotions occur in the future. At the same time, as a repetitive behavior, the self-reinforcing nature of NSSI leads individuals to adopt it as an avoidance strategy over time, thus possessing the characteristics of addiction, which poses challenges to terminating such behaviors. Therefore, when adolescents derive a sense of pleasure, transient psychological stimulation, or relief from unpleasant emotions through NSSI, they are likely to routinely use it. In clinical treatment, it is crucial that adolescents are provided with outlets or other methods for expressing their feelings. Elucidating the motivations, psychological challenges, and nursing needs of adolescents with NSSI is paramount to assisting adolescents in reducing and ceasing NSSI behaviors.

#### 4.2.2 Medication compliance affects NSSI risk

From a pharmacological standpoint, drug compliance refers to the degree to which a patient implements the drug treatment plan. Deviations from the medication requirements of the treatment plan in any aspect of this process due to patient-related factors lead to varying degrees of non-compliance, which eventually affects therapeutic outcomes. Data on 2,013 adolescents were obtained from the Canada Mental Health Reporting System using the Resident Assessment Instrument-Mental Health (RAI-MH) and analyzed using logistic regression. According to the results, intentional abuse of prescription drugs was identified as the most significant factor related to NSSI (43). Following hospital discharge and during the out-of-hospital follow-up period, healthcare professionals should focus on providing important compliance guidance and intervention, emphasizing the safety of medications and the ability of regular medication adherence to facilitate recovery and prevent recurrence.

#### 4.2.3 NSSI history is a key factor in predicting NSSI

A history of NSSI may result in increased pain tolerance and decreased fear of death, culminating in recurrent self-harming behaviors (44, 45). In addition, a huge body of evidence indicates that patients with a history of NSSI are more likely to struggle with emotional management and self-efficacy resistance. Difficulties in emotion management and resistance to self-efficacy influence the associations between outcome expectations and previous NSSI history (46). Furthermore, an adolescent’s previous suicide attempt can easily escalate into additional self-harm attempts. However, when adolescents engage in self-harming behavior, they frequently refrain from additional harm out of the associated fear and pain (47). This inherent urge for self-resistance and self-injury mostly results in NSSI. Taken together, challenges linked to emotional regulation and low self-efficacy in resisting self-harm are indicative of a history of NSSI. Addressing the patient’s emotional control, self-efficacy, and underlying causes of NSSI can successfully prevent recurrences.

#### 4.2.4 Impulsive adolescents are more likely to develop NSSI

According to recent research, NSSI is associated with impulsive tendencies but not with impulsive actions noted in laboratory tasks, even in situations involving negative moods (14). Neurocognitive impulsivity is connected with more frequent and recent NSSI episodes, particularly in negative emotional circumstances, including actual or perceived criticism in close relationships. NSSI is a method for promptly releasing negative emotions and anger. However, an earlier study identified an association between recurrent NSSI and greater behavioral compulsivity and poor decision-making, but not with behavioral impulsivity (48), inconsistent with the findings of this research.

The use of impulsive qualities as predictors of NSSI requires the incorporation of other laboratory markers. To determine the association between impulsive behavior and NSSI, longer follow-up periods or larger sample sizes are required. As key contributors to the emotional support network of hospitalized adolescents with NSSI, healthcare professionals can aid adolescents in identifying and articulating specific emotions and feelings about NSSI and engaging in self-awareness and cognitive reconstruction. They can also guide adolescents to accept their negative emotions in a healthy manner and focus on the present, thereby effectively reducing the risk of impulsive events.

#### 4.2.5 Lower pain and belief perception beliefs scores were associated with a higher risk of NSSI

In recent years, an increasing number of studies have examined alterations in the pain perception of patients with NSSI. Adolescents with impaired pain perception may adopt negative coping mechanisms in response to pain, leading to a reduction in pain levels during NSSI episodes and an increase in the frequency and severity of NSSI. More importantly, individuals with NSSI may perceive pain differently from non-NSSI patients. Kirtley et al. (49) demonstrated that patients with NSSI experience altered pain perception. While studies exploring the relationship between NSSI and pain perception are scarce, several studies have concluded that the pain threshold and pain tolerance of patients with NSSI are higher (50, 51). For example, Schmahl et al. (52) determined that painful stimulation promoted the activation of the dorsal prefrontal cortex in patients with NSSI and concomitantly inhibited the activation of the posterior parietal cortex compared to the healthy control group. Changes in pain perception in response to this unpleasant stimulation may represent a resistance mechanism in NSSI patients (53). Furthermore, post-hospital education on pain perception and coping style is critical. Adolescents lack positive coping methods for psychological pain and physical pain. Their negative coping style leads to a cycle of self-denial and self-harm, which drives NSSI behaviors. Understanding and enhancing their pain coping mechanisms can assist in mitigating NSSI behaviors.

#### 4.2.6 Worse sleep quality was positively correlated with the risk of NSSI

Insomnia increases anxiety levels, which in turn exacerbates NSSI behavior. Poor sleep duration and insomnia symptoms have been established to enhance an individual’s impulsivity, which is a significant risk factor for NSSI (54). Specifically, sleep disorders and poor sleep quality decrease the ability to regulate emotions, thereby increasing the risk of NSSI behavior (55, 56). Herein, 81.2% of participants scored in the clinical range for poor sleep on the PSQI. Likewise, 81.2% reported a circadian preference for evening (night owl) patterns. PSQI scores were positively associated with the levels of self-harm (suicide attempts and NSSI) and were identified as a predictor for self-harm within 30 days. Of note, the rates of self-harm were high during the follow-up period, with 45.0% and 33.7% at 6 and 12 months, respectively (54). The current study uncovered that sleep quality was strongly correlated with NSSI, in agreement with the findings of previous studies. In order to reduce the risk of NSSI, healthcare practitioners or parents address sleep disorders in adolescents, encourage regular sleep schedules, and ensure appropriate sleep on weekends. In addition, psychological interventions for college students with NSSI could incorporate psychological counseling or treatment methods (such as cognitive behavioral therapy for insomnia and stimulation control therapy) to alleviate sleep disorders, thereby indirectly mitigating NSSI behaviors.

### 4.3 The NSSI risk prediction model displayed high predictive performance

Based on multivariate regression analysis, a nomogram integrating multiple predictors was developed and validated (57). The area under the ROC curve of the original model was 0.9989, with a 95% confidence interval of (0.9979, 0.9999). Following this, the clinical information of new patients was introduced into the model for risk prediction, yielding an area under the ROC curve of 0.9703, with a 95% confidence interval of (0.9167, 0.9999), signifying that the NSSI risk prediction model could accurately predict the risk of NSSI within 6 months after discharge. Moreover, despite participants in the modeling group and the validation group being from different hospitals, the area under the ROC curve was comparable, highlighting the generalizability of the model. Therefore, this model can be used to predict NSSI behaviors within 6 months following hospitalization.

### 4.4 Practical implications and research on the NSSI risk prediction model

The developed models may enhance the decision-making process of physicians regarding patient adherence to the identified risk factors. The risk score model generated herein, based on the identified major risk factors, allows for the collection of information using fewer variables, positioning it as a simple and effective approach. When applying this model to screen high-risk groups, healthcare professionals can select appropriate cut-off points tailored to their clinical needs. Providing real-time predictions of NSSI risk factors during patient enrollment may enable healthcare professionals to proactively implement intervention strategies and prioritize high-risk patients. For adolescents identified as high-risk during follow-up, urgent psychological assistance should be administered. Furthermore, the medical staff should contact the parents and instruct them to monitor their child’s sleep, ensure medication adherence, and assist them in overcoming negative feelings. Overall, this study laid a theoretical and methodological groundwork for future longitudinal cohort studies focusing on teenagers. Nevertheless, it is worth acknowledging that the NSSI prediction model remains in the early stages of development. While this study primarily focused on identifying key risk factors, future reports will describe how we also used study data to develop and validate an adaptive screening model. Theoretically, this research has established the groundwork for a more robust, comprehensive, and scientific model.

## 5 Limitations

Nonetheless, this study has some limitations that cannot be overlooked. To begin, short and customized measures were employed to evaluate the majority of NSSI components in order to minimize respondent burden. Despite the assessed variables being identified as significant univariable predictors of NSSI, the use of brief scales may have compromised the reliability of the measurements and our ability to properly capture each construct. To identify future predictors while limiting patient impact and the workload on the medical personnel, additional objective indicators should be included. In addition, given the cross-sectional nature of this study, the acquired data may be biased, posing challenges in inferring causality. The area under the ROC curve, sensitivity, and specificity of the risk assessment model are not optimal, indicating the need for future studies to identify more significant explanatory factors. Expanding model generalizability necessitates the incorporation of new data sources, especially from recurrent patients. Third, we did not adjust for non-response by weighting the sample. Further analysis of indicator weights may be more conducive to improving the predictability of the model. Therefore, ongoing investigation into risk variables is vital for further development of this model.

## 6 Conclusion

In this study, the number of relapses, medication adherence, and NSSI history were identified as the most significant predictors of patient outcomes. Despite the limitations posed by the variability of risk variables for risk stratification, the results conjointly indicate that a multivariable prediction model can be beneficial for the short-term prediction of NSSI in adolescents (58). Additionally, the predictive model constructed herein exhibited favorably discriminatory power and accuracy and could effectively predict the risk of NSSI in adolescents. More importantly, these models may assist in identifying potentially key targets for clinical risk evaluation and prevention. Using prediction algorithms derived from large-scale data sources, screening systems for risk recognition may be pioneered (58). To enhance the validation of clinical samples, modern tools or machine learning techniques based on AI can be used.

## Data Availability

The original contributions presented in the study are included in the article/supplementary material. Further inquiries can be directed to the corresponding author.
